# Variations of bile bacterial community alongside gallstone disease progression and key taxa involved in poor outcomes after endoscopic surgery

**DOI:** 10.1186/s40001-023-01308-y

**Published:** 2023-09-02

**Authors:** Xunchao Cai, Yao Peng, Yajie Gong, Xiuting Huang, Lu Liu, Yifan Chen, Jingfeng Du, Zhongming Dai, Yun Qian, Long Xu

**Affiliations:** 1 Department of Gastroenterology and Hepatology, Shenzhen University General Hospital, Shenzhen, 518055 China; 2 Marshall Laboratory of Biomedical Engineering, Shenzhen University, Shenzhen, 518055 China

**Keywords:** Biliary disease, Bacterial translocation, Intestinal bacterial infection, Oral cavity bacteria, Taxa presence, Clinical index, Common bile duct

## Abstract

Gallstone disease is a prevalent biliary disease worldwide, and bacteria play vital roles in the disease development and progression, as well as the prognosis after endoscopic surgery. However, there have been limited studies to explore the key taxa involved. In this study, bile samples from healthy controls (HCs, liver donors without hepatobiliary disease) and three diseased groups, namely patients with gallbladder stones (GBS), patients with common bile duct stones (CBDS), and patients with stricture in the common bile duct (SCBD), were collected and analyzed. Bacterial community characterization based on 16S rRNA amplicon sequencing showed that bacterial diversities did not change significantly alongside gallstone disease development and progression. The predominant phyla in each group were Proteobacteria, Firmicutes, Bacteroidota, and Fusobacteriota, representing over 80% in abundance of the biliary bacteria community. Specifically, the abundance of Proteobacteria decreased greatly while that of Firmicutes and Bacteroidota increased greatly in the diseased groups when compared to that in HCs. Moreover, linear discriminant analysis identified several genera highly represented in the diseased groups. Among them, *Klebsiella*, *Prevotella*, *Pseudomonas* and *Veillonella* are persistent in both the HCs group and the diseased groups, indicating an enrichment of local bile bacteria in the diseased bile; while *Lachnoanerobaculum*, *Atopobium*, *Oribacterium*, and *Stomatobaculum*, those aligned to oral cavity taxa, are persistent in the diseased groups but are transient in the HCs group, and their abundances sequentially increased with the disease development and progression (HCs→GBS→CBDS→SCBD), implying a translocation and colonization of the oral cavity bacteria in the diseased bile. Moreover, co-occurrence network analysis revealed that bacterial infection (e.g., *Photobacterium* and *Plesiomonas*) from the intestine was developed during endoscopic surgery with reduced bile bacteria diversity. The results of this study revealed that the bile bacterial community is relatively stable and dominated by a few persistent taxa. Moreover, we hypothesized that translocation and colonization of specific bacteria from the oral cavity happens alongside gallstone disease development and progression, and bacterial infection from the intestinal tract results in poor outcomes after endoscopic surgery.

**Supplementary Information:**

The online version contains supplementary material available at 10.1186/s40001-023-01308-y.

## Introduction

Gallstone disease is a prevalent cause of hospitalization in many regions of the world, which may occur at any location of the gallbladder and biliary tract [1]. Certain ethnic groups develop gallstone disease more frequently than others [2]. For example, approximately 10–15% of the American adult population (20–25 million) develops gallstone disease [3–5], while only 3% of the adult population develops the disease in many parts of Africa [6]. It has been observed that gallstone disease annually contributes to the highest medical equipment costs ($4.3 billion) and the second highest physician costs ($0.75 billion) among all digestive diseases [7, 8]. Although approximately 80% of people with gallstones in the biliary system present asymptomatic [2, 9], acute inflammation caused by this disease results in a strong cramp-like visceral pain in the right upper abdomen [10]. Other complications include gallbladder inflammation (cholecystitis), pancreas inflammation (pancreatitis), jaundice obstruction, and bile duct infection (cholangitis) [10]. Specifically, gallstones in the gallbladder (i.e., gallbladder stones, GBS) can often progress and migrate into the common bile duct, causing the formation of common bile duct stones (CBDS). Furthermore, the chronic inflammation caused by gallstone disease may progress to malignant biliary diseases such as gallbladder cancer and bile duct cancer [11].

In recent years, microbiota, especially bacteria, have been recognized as vital environmental factors that are associated with gallstone disease and its complications while being related to the progression of the disease [8, 12]. For example, bacterial infection with gallstone disease causes chronic inflammation in the biliary system, which has been acknowledged as a risk factor for the development of bile duct cancer (i.e., cholangiocarcinoma) [13, 14] and has been linked to a high incidence of gallbladder cancer [11, 15]. Consequently, knowledge of bacterial community dynamics in the bile of gallstone patients and healthy people may help prevent the development of gallstone diseases and associated complications. Currently, the microbial residents of the gut tract and some biological fluids in different locations of our body have been extensively studied, and their correlations to the pathological state of the host have been well recognized during the last two decades; however, such studies on bile are limited [16, 17]. Enterobacteria have been documented as frequently isolated from the bile fluids of cholelithiasis patients, and have been suggested to contribute to gallstone formation [18]. Very recently, high-throughput sequencing technology revealed that Enterobacteriaceae were abundant in the biliary tract of patients with acute cholecystitis and gallstones [19, 20]. In addition, the genera *Prevotella*, *Streptococcus*, *Veillonella*, *Fusobacterium*, and *Haemophilus* are prevalent in the bile ducts of patients with primary sclerosing cholangitis [21]. These previous findings demonstrated that different bacterial communities were present in diseased biliary systems, which may correlate with the etiology of the disease. Some studies discovered biliary disease-enriched bacteria—such as Enterobacteriaceae, Bacterioidaceae, Prevotellaceae, Porphyromonadaceae, and Veillonellaceae—in chronic cholecystitis and gallbladder cancer [22, 23]. Nevertheless, to date, only a few studies have explored the bile bacterial communities in individuals without any bile or liver-related disorders [22, 23]. Furthermore, the bacterial assemblage variations in gallstone disease formation and progression (i.e., Healthy Controls (HCs)→GBS→ CBDS→Stricture of the Common Bile Duct (SCBD)) have not yet been studied.

For the treatment of gallstone diseases, the gallstones in GBS and CBDS patients can be removed using endoscopic approaches, such as natural orifice transluminal endoscopic surgery (NOTES) and endoscopic retrograde cholangiopancreatography (ERCP), respectively [24]. Bacteremia and acute pancreatitis are potential complications, particularly in patients who have previously undergone ERCP, and the bile microbiota have been suggested to be contaminated with the duodenal microbiota during the procedure [25], whereas this has not been studied for NOTES. Furthermore, associations between bile bacteria and the clinical indexes of specific biliary diseases remain under-studied.

In this study, bile samples from healthy liver donors and gallstone patients at different stages who have undergone ERCP or NOTES were collected sterilely, and 16S rRNA amplicon sequencing was performed to determine the bile bacterial community variations between these different pathological states to find the key taxa involved in these diseases or endoscopic surgery treatments. Moreover, the correlation between bacteria and clinical indexes was explored and discussed to identify the bacteria that may be involved in post-endoscopic complications.

## Materials and methods

### Participant enrollment and sample collection

All participants, namely GBS patients, CBDS patients, SCBD patients, and HCs were enrolled by the Department of Gastroenterology and Hepatology, Shenzhen University General Hospital (SUGH), Shenzhen University, from January 2019 to September 2021. All of them were ethnically Han Chinese originating from the southern region of China, adhering to a traditional Chinese dietary pattern and had not been on long-term medication. Briefly, the abdominal computed tomography (CT) was used to diagnose all patients. Moreover, the magnetic resonance imaging (MRI) was further used to diagnose SCBD patients. Participants in GBS and CBDS were diagnosed with chronic cholecystitis and cholangitis, respectively. Participants in SCBD were patients with gallstone and stricture in the common bile duct, who were diagnosed with malignant diseases, and comprised six cholangiocarcinoma and two pancreatic head carcinoma patients. HCs were individuals without hepatobiliary disease, but were liver donors who had suffered a brain accident or stroke (not more than a 24 h stay in the hospital’s Intensive Care Unit (ICU)). The study was approved by the Ethics Committee of the SUGH, Shenzhen University, and written informed consent was obtained from all participants before enrollment. In addition, sample collection from the participants was approved by the Research Ethics Board of the SUGH, Shenzhen University. The enrolled patients comprised those diagnosed with CBDS and SCBD and have undergone ERCP, and those diagnosed with GBS and have undergone NOTES. All enrolled patients were administered intravenous ciprofloxacin (400 mg Intravenous Injection (IV) drip), one hour before endoscopic surgery. Sterile saline was used to wash the devise root of endoscope and cannulation tube when performing the surgery. Approximately five to twenty milliliters of bile fluid were aspirated sterilely, immediately placed in germ-free sputum cups, and stored at − 80 °C until required. The inclusion criteria comprised (1) age ranged 18–80, (2) patients with symptomatic gallstone diseases or SCBD. The exclusion criteria comprised (1) patients that were unable to provide informed consent, (2) the presence of biliary tract or liver disease comorbidities not belonging to the enrolled group, (3) having a history of ERCP, NOTES, or other biliary surgeries, (4) participants get antibiotic therapy before bile sampling within the previous three months, and (5) participants with asymptomatic stones. The clinical indexes detected in this study include white blood cell (WBC), C-reactive protein (CRP), aspartate aminotransferase (AST), alanine aminotransferase (ALT), alkaline phosphatase, procalcitonin (PCT), amylase activity (AMY), and lipase activity (LIPA), which were performed by the Department of Clinical Laboratory at the SUGH using standard methods. For the clinical indexes of the patients, AMY, LIPA, and PCT were measured within 24 h after the endoscopic surgery, while other indexes were measured before the endoscopic surgery within 48 h.

### *DNA extraction, sequencing*,* and taxa identification*

Solid residues (e.g., bacteria, cell debris, and micro-gallstones) in a 5 mL bile sample were collected through centrifugation at 12,000 × *g* and 4 °C for 10 min. The collected pellets were then completely resuspended in 100 μL of TE buffer using vortex shaking. The total DNA was then extracted using the E.Z.N.A.® Bacterial DNA Kit D3350-02 (Omega Bio-tek, Norcross, GA, USA) with the full procedures according to the manufacturer’s instructions. DNA quality/purity was robustly determined using the NanoDrop™ ND-1000 Spectrophotometer (Thermo, Massachusetts, USA). Twenty microliter quality-controlled DNA (criteria: total DNA amount ≥ 0.15 μg, DNA concentration ≥ 10 ng/μL and OD260/OD280 = 1.8–2.0) was outsourced to Novogene Company (Nanjing, China) to construct 16S rRNA (V3-V4) sequencing libraries, to perform sequencing, and to conduct standard QIIME2 pipeline analyses. Briefly, the primers for the V3-V4 fragment amplification were 5’-CCTACGGRRBGCASCAGKVRVGAAT-3’ and 5’-GGACTACNVGGGTWTCTAATCC-3’. PCR reactions were carried out in a mixture with 15 μL of 2 × Phusion® High-Fidelity PCR Master Mix (New England Biolabs); 0.2 μM of forward and reverse primers, 30 ng of template DNA, followed by the proper amount of deionized water to make the mixture a final volume of 30 μL. Thermal cycling was performed with the initial denaturation at 98 °C for 1 min, followed by 30 cycles of denaturation at 98 °C for 10 s, annealing at 50 °C for 30 s, and elongation at 72 °C for 30 s. Finally, it was kept at 72 °C for 5 min. The PCR products were detected using electrophoresis on 2% agarose gel. Next, the mixture of PCR products was purified using the Qiagen Gel Extraction Kit (Qiagen, Germany). The sample-specific barcodes were then added to the purified PCR products and ligated with the adaptor to construct sequencing libraries using a TruSeq® DNA PCR-Free Sample Preparation Kit (Illumina, USA) according to the manufacturer's recommendations. The library quality was assessed using the Qubit® 2.0 Fluorometer (Thermo Scientific) and the Agilent Bioanalyzer 2100 system. Sequencing was performed using the PE250 strategy on the Illumina NovaSeq platform. QIIME2 (version 2021.2) was used to filter raw reads with default parameters and split the samples based on their unique barcode sequences. The DADA2 software implanted in QIIME2 was used to denoise the data and produce amplicon sequence variants (ASVs). The taxonomic annotation was performed based on the “silva-138-99-nb-classifier” pretrained in QIIME2. Further data visualization and analyses were performed using R (v4.1.2) by the authors.

### Characterization of bacterial communities

The following analyses, including alpha diversity and beta diversity, were performed at the ASV level using QIIME2 (version 2021.2) with default parameters. Briefly, alpha diversity was represented using both the Chao1 index for ASV richness and the Shannon–Wiener index for within-sample diversity [26]. Beta diversity was represented using weighted UniFrac distances and visualized using principal coordinate analysis. Spearman’s ranked correlation method (999 permutations) was used to determine the correlations between the alpha-diversity and clinical indexes, and significant correlation was defined according to the method described by Cohen (1988) [27] as follows (*p*-value ≤ 0.05): small correlation (coefficient 0.1–0.3), medium correlation (coefficient 0.3–0.5), strong correlation (coefficient ≥ 0.5). The two-sided Welch’s test was used to determine the taxonomic abundance differences between the different groups. Unless stated, a *p*-value ≤ 0.05 was defined as significant. In most natural ecosystems, the core taxa are persistent and high in abundance, while the satellite species are transient and low in abundance [28]. To better understand the bacterial assemblages in the bile, we categorized the bacterial community into the following three ecological categories based on the occurrence frequency: persistent (detected in ≥ 75% of samples), intermittent (detected in 25–75% of samples), and transient (detected in ≤ 25% of samples) [29]. The biomarker taxa that most likely explained the differences between the different groups (i.e., GBS, SCBD, CBDS, and HCs) were determined using linear discriminant analysis (LDA) effect size (LEfSe). Taxa with an LDA score higher than 2.0 were determined to be significant.

### Correlation network analysis between the bacteria and the clinical indexes

Weighted correlation network analysis (WGCNA) was performed to find highly correlated taxa clusters/modules using the R software package "WGCNA" to relate the bacterial groups to one another, and the clinical indexes, according to the software manuals (https://horvath.geneticsucla.edu/html/CoexpressionNetwork/Rpackages/WGCNA/) [30]. In detail, the taxa abundance matrix was first clustered using the hierarchical clustering function implanted in WGCNA to check if there were any outliers, which would be removed in further analysis. Finally, the dynamic tree cut method was used to identify the co-occurrence taxa modules in the whole bacterial community (minModuleSize = 30 and mergeCutHeight = 0.25). Then, Cytoscape v3.7.1 was used for network visualization and topological analysis [31].

## Results

### Cohort characteristics

To detect the specific biliary bacterial communities in the gallstone patients, 62 participants were enrolled in this study, comprising 31 CBDS patients, 15 GBS patients, 8 SCBD patients, and 8 liver donors without hepatobiliary disease (HCs). The demographic and clinical characteristics of the 54 patients with biliary diseases are listed in Table 1. Collectively, participants from the disease groups (i.e., GBS, CBDS, SCBD) displayed poor liver situations when compared to those of HCs, indicated by higher total bilirubin (TBIL), alanine transaminase (ALT), aspartate transaminase (AST), or alkaline phosphatase (ALP) indexes (Kruskal–Wallis test, *p* ≤ 0.05) (Table 1). However, liver situations between disease groups were not significant different by most of the indexes, except that the ALT in CBDS is significantly higher than the others. The inflammatory index LIPA in the circulation serum after endoscopic surgery were significantly higher in the disease groups than in the HCs, indicating a risk of inflammatory after endoscopic surgery. However, these differences are smaller or diminished between the disease groups (Table 1). Although clinical indicator PCT of microbial infection was not significant different between the disease groups and HCs, we observed great increasing of PCT in some the patients. These results suggested that acute inflammation commonly appeared after endoscopic surgery, while the microbial infection may also appear at a low risk.

**Table 1:** The demographic characteristics of the enrolled patients

﻿Characteristics	HCs	GBS	CBDS	SCBD
Patients, n	8	15	31	8
Female, n (%)	3, (37.50)	6, (40.00)	14, (45.16)	6, (75.00)
Age, years	32.37 ± 10.34	39.71 ± 12.37	56.03 ± 16.97	56.63 ± 8.54
BMI, kg/m^2^	21.85 ± 2.13	25.49 ± 4.28	23.73 ± 3.86	21.25 ± 3.48
ERCP	NA	NA	YES	YES
NOTES	NA	YES	NA	NA
TBIL, µmol/L	13.30 ± 4.27^a^	29.33 ± 52.27^ab^	62.06 ± 55.08^ab^	123.66 ± 89.8^bc^
ALT, U/L	23.38 ± 19.89^a^	54.86 ± 56.69^ab^	200.07 ± 256.14^c^	147 ± 202.55^bc^
AST, U/L	20.75 ± 7.55^a^	39.75 ± 42.51^ab^	130.68 ± 238.69^bc^	81.63 ± 71.27^ab^
ALP, U/L	66.38 ± 13.11^a^	120.69 ± 118.7^ab^	249.62 ± 226.98^bc^	214.16 ± 107.60^ab^
CRP, mg/L	51.51 ± 32.80^b^	28.30 ± 69.75^a^	35.63 ± 52.81^a^	22.78 ± 23.56^a^
WBC, 10^9^/L	9.63 ± 4.82^a^	6.94 ± 2.40^a^	8.28 ± 5.05^a^	8.09 ± 5.12^a^
PCT, ng/mL	0.43 ± 0.40^a^	0.41 ± 0.55^a^	3.57 ± 7.63^a^	0.53 ± 0.58^a^
AMY, U/L	56.88 ± 9.39^a^	301.88 ± 222.5^a^	266.29 ± 258.30^a^	240.25 ± 302.42^a^
LIPA, U/L	86.00 ± 26.95^a^	120.69 ± 118.8^b^	1980.37 ± 3156.8^bc^	1994.25 ± 3354.6^bc^

For participants in the diseased groups, PCT, AMY and LIPA were measured after the endoscopic surgery, the others were measured before the endoscopic surgery. NA denotes data not applicable. HCs, GBS, CBDS and SCBD represent Healthy Controls (liver donors without hepatobiliary disease), patients with Gall-Bladder Stones, patients with Common Bile Duct Stones, and patients with Stricture in the Common Bile Duct, respectively. BMI, TBIL, ALT, AST, ALP, CRP, WBC, PCT and AMY represent body mass index, total bilirubin, alanine transaminase, aspartate transaminase, alkaline phosphatase, C-reactive protein, white blood cell, procalcitonin and amylase, respectively. Kruskal–Wallis test was adopted to evaluate the differences of clinical index between group, *p* ≤ 0.05 was considered significant

#### Low variations in biliary bacterial assemblages under different pathological states

**Fig. 1:**
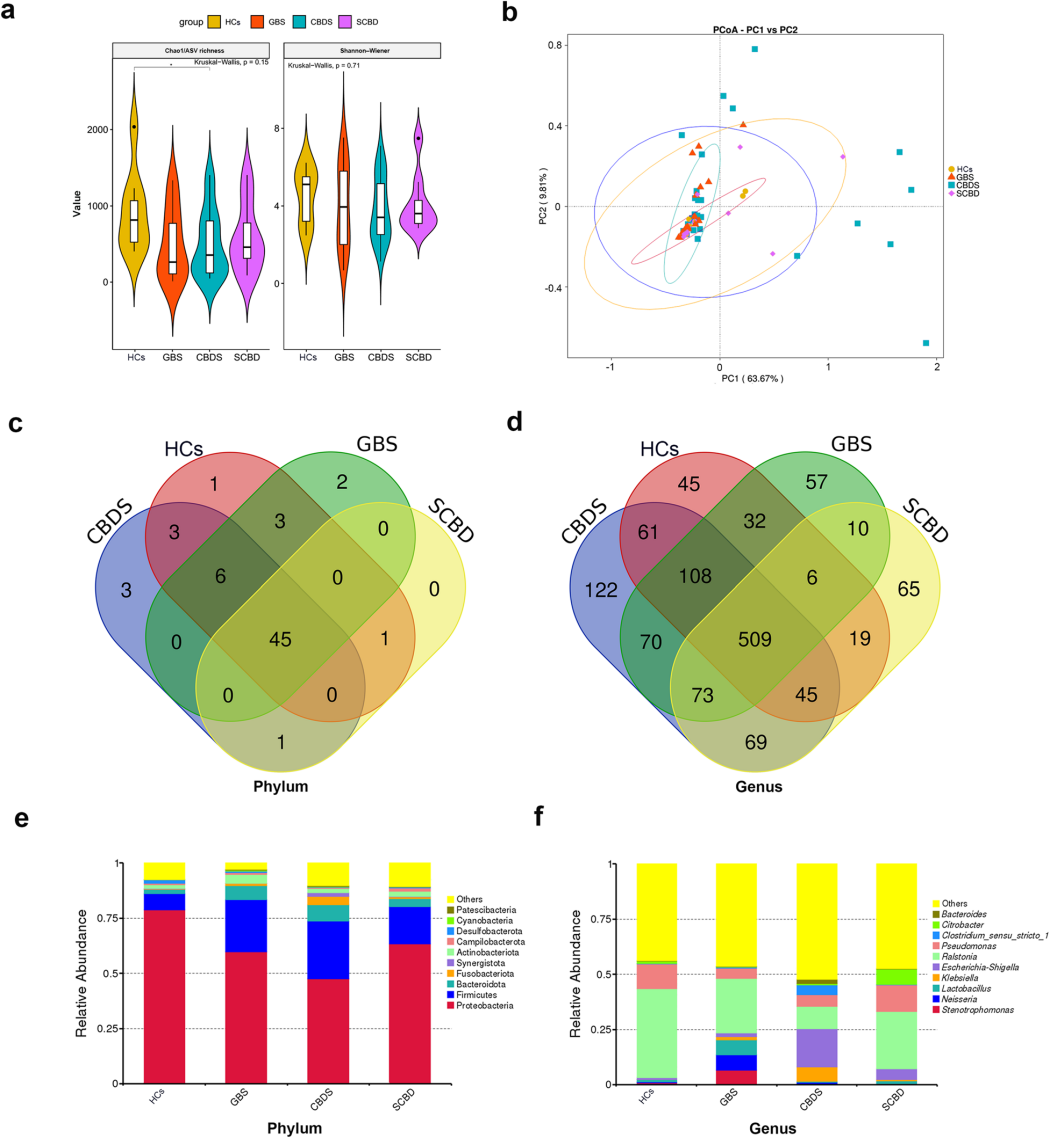
Global view of biliary bacterial assemblages in different pathological states relevant to gallstone disease. HCs, GBS, CBDS and SCBD represent Healthy Controls (liver donors without hepatobiliary disease), patients with Gall-Bladder Stones, patients with Common Bile Duct Stones, and patients with Stricture in the Common Bile Duct, respectively. **a** Alpha diversity between groups was measured by the Shannon–Weiner and Chao1 indexes, representing within-sample diversity and ASV richness, respectively. Asterisk “*” represents that significant difference exist between groups. (Kruskal–Wallis test, *p* ≤ 0.05) **b** Beta-diversity is indicated using principal coordinate analysis based on weighted UniFrac distances; eclipse represents the 95% confidence interval. (**c**), **d** Venn diagrams displaying unique and shared taxa between different groups at the phylum and genus levels respectively. **e**, **f** Bar plots showing the cumulative abundance of the top ten taxa in each group at the phylum and genus levels

Before analyzing the sequencing data, quality control was conducted to remove low quality sequences or chimera sequences. Summary of the quality-controlled data is listed in Additional file 1: Table S1. Furthermore, we confirmed that the sequencing depth was adequate to represent the bacterial assemblages in each sample by checking the alpha rarefaction curve based on Shannon diversity and accumulated ASVs (Additional file 2: Fig. S1). From a global view, the alpha diversity, indicated by the Shannon–Weiner index and Chao1 index, among all the tested groups showed no significant differences in diversity and ASV richness (Kruskal–Wallis test, *p* = 0.15) (Fig. 1a). Pairwise comparisons of the Chao1 indexes between HCs and CBDS showed that the ASV richness in the CBDS patients decreased significantly compared with that in HCs (Fig. 1a). For beta-diversity, no significant differences were observed among all groups (analysis Anosim, *p* = 0.776) (Fig. 1b). However, a much higher heterogeneity was observed within the CBDS and SCBD groups based on UniFrac distances (Fig. 1b), suggesting that the bacteria community in the diseased gallbladder was less stable when taking the bacteria phylogeny into account. A Venn diagram showed that the bile maintained a very stable core biliary microbiota at the phylum level between the patients (Fig. 1c) and HCs. At the genus level, the CBDS patients had the most while the HCs had the least number of unique taxa (Fig. 1d).

In detail, the most abundant phyla in the bile were Proteobacteria, Firmicutes, Bacteroidota, Fusobacteriota, Synergistota, Actinobacteriota, Campilobacterota, Desulfobacterota, Cyanobacteria, and Patescibacteria (Fig. 1e). Among them, the abundance of Proteobacteria decreased sequentially in HCs (78.86%), SCBD (63.40%), GBS (59.2%), and CBDS (47.41%), while Firmicutes and Bacteroidota increased sequentially (Fig. 1e). The most abundant genera were *Stenotrophomonas*, *Neisseria*, *Lactobacillus*, *Klebsiella*, *Escherichia-Shigella*, *Ralstonia*, *Pseudomonas*, *Clostridium_sensu_stricto_1*, *Citrobacter*, and *Bacteroides*, among which *Ralstonia* decreased sequentially among the HCs (40.33%), SCBD (25.90%), GBS (24.73%), and CBDS (10.15%) (Fig. 1f). Noticeably, the abundance of *Escherichia-Shigella* (17.27%) in CBDS was tenfold, threefold, and 20-fold higher than that in GBS (1.55%), SCBD (4.93%), and HCs (0.81%), respectively (Fig. 1f). Taken together, the results show that bile harbors a very stable bacterial community, while specific taxa alterations can be observed in the diseasing states, particularly in patients with common bile duct stones.

### Exponential model between occurrence frequency and average abundance of biliary bacteria

**Fig. 2:**
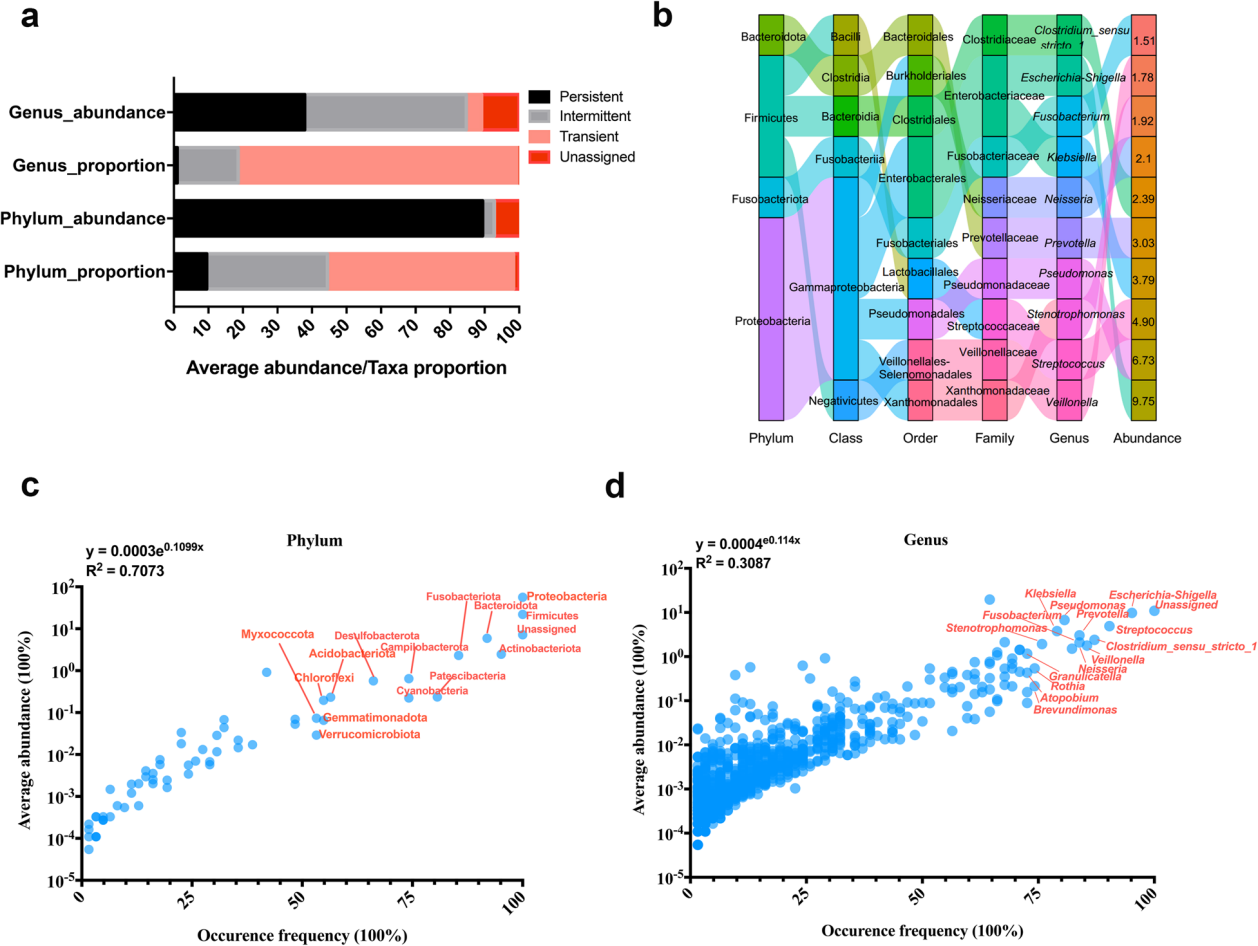
Characterization of the biliary bacterial assemblage patterns based on taxa abundance and occurrence frequency. **a** Taxa category defined by the occurrence frequency, their proportion to all detected taxa, and their average abundance at the phylum or genus levels. **b** Taxonomic hierarchy of persistent genera in the biliary microbiota. **c**, **d** The best-fitted model for average taxa abundance and occurrence frequency of the biliary bacteria at the phylum and genus levels, respectively. Persistent taxa are those detected in most samples (i.e., ≥ 75 samples), which are most probably local organisms; intermittent taxa are those detected in medium proportion of samples (i.e., 25–75% samples), which are probably translocated and colonized organisms in specific state; transient taxa are those detected only in a small number of samples (i.e., ≤ 25 samples), which are probably contaminated or translocated organisms

As the biliary bacteria community stays relatively stable under different pathological states, we explored the taxa occurrence frequency and average abundance for a global view of the biliary bacterial assemblage patterns. Six phyla, namely Proteobacteria, Firmicutes, Actinobacteriota, Bacteroidota, Fusobacteriota, and Patescibacteria were classified as persistent phyla (i.e., detected in over 75% samples). Although they represented only 9.52% (6/63) proportion of all the detected phyla in the bile, the accumulated average abundance of these phyla was 89.41% (Fig. 2a). Notably, abundances of the unassigned taxa at the phylum and genus levels were 7.17% and 10.89% respectively (Fig. 2a), indicating a decreased resolution of bacterial assignment at the lower taxonomic level. Ten genera, namely *Escherichia-Shigella*, *Streptococcus*, *Clostridium_sensu_stricto_1*, *Veillonella*, *Neisseria*, *Prevotella*, *Fusobacterium*, *Pseudomonas*, *Klebsiella*, and *Stenotrophomonas*, were classified as persistent genera (Fig. 2b), which represented 0.99% (10/1014) of all the detected genera and the accumulated abundance of these genera was 37.90% (Fig. 2a). Detailed taxonomic classification at different levels displayed that half of the persistent genera belonged to the phylum Proteobacteria, including *Escherichia-Shigella*, *Pseudomonas*, *Klebsiella*, *Neisseria*, and *Stenotrophomonas* (Fig. 2b). Overall, the minority bacteria (i.e., persistent taxa) in the bile microbiota occupied dominant positions. The correlations between the taxa abundance and occurrence frequency were further found to be best fitted for the exponential formula (Fig. 2c, d), especially at the phylum level (*R*^2^ = 0.707), which showed a strong exponential correlation, and the minority taxa accounted for most of the abundance in the entire community (Fig. 2c, d).

### Highly represented biliary bacteria under the different pathological states

**Fig. 3:**
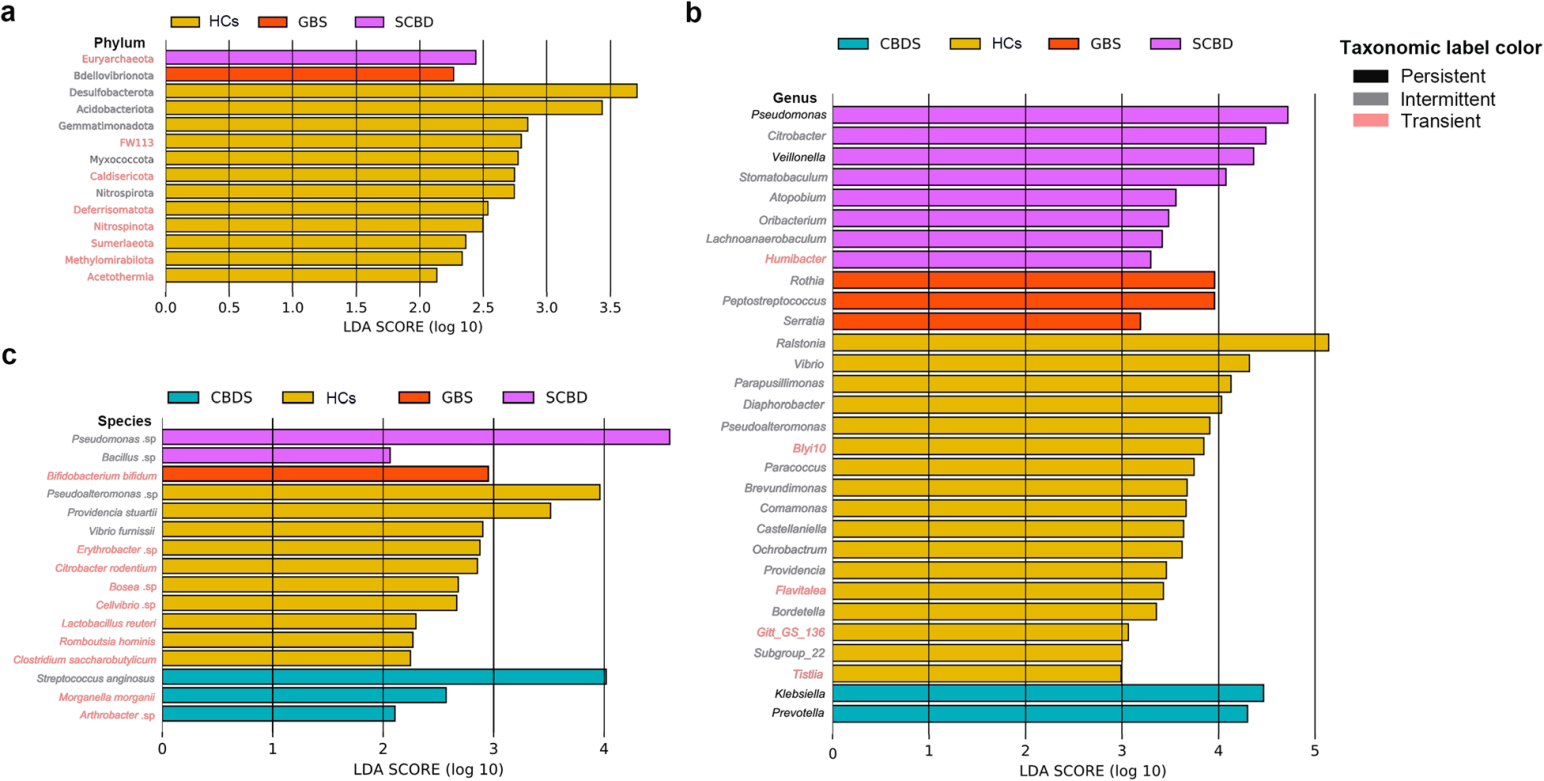
Histograms of the linear discriminant analysis (LDA) scores featured taxa at the phylum (**a**), genus (**b**), and species (**c**) levels in the GBS, SCBD, CBDS, and HCs groups. HCs, GBS, CBDS and SCBD represent Healthy Controls (liver donors without hepatobiliary disease), patients with Gall-Bladder Stones, patients with Common Bile Duct Stones, and patients with Stricture in the Common Bile Duct, respectively. Taxa with LDA scores (log 10) > 2 and the top 30 ranked are displayed

To identify specific biliary taxa represented in the healthy or diseased groups, LEfSe analysis was conducted, and the taxa with an LDA score cutoff of 2.0 were considered significant, and only the top 30 ranked taxa were displayed in the figure (Fig. 3). No featured phylum was found in the CBDS group, and only one taxon was featured in either the GBS or SCBD groups (Fig. 3a). Most of the highly represented taxa in each group were transient or intermittent taxa, suggesting that specific taxa may be commonly present or absent between different pathological states (Fig. 3). At the genus level, the only highly represented taxa in CBDS are two persistent taxa (i.e., *Klebsiella* and *Prevotella*). Moreover, two persistent genera (i.e., *Pseudomonas* and *Veillonella*) were highly represented in SCBD (Fig. 3b), and the number of highly represented genera in the SCBD group is much higher than that in the GBS and CBDS groups (Fig. 3b). At the species level, most of the highly represented taxa in each group are transient species (Fig. 3c), which is due to the low resolution of the V3-V4 16S rRNA on bile bacteria species, causing the poor taxonomic assignment of bacterial taxa at the species level.

The detailed occurrence frequencies of these featured genera are listed in Table 2. Among them, *BIyi10*, *Flavitalea*, *Gitt_GS_136*, *Humibacter*, and *Tistlia* had a low possibility of making contributions to the transformation of health or disease states in the biliary system, as they were presented in less than 25% samples of each group and had very low abundance. Some other genera that were transient or intermittent in HCs but persistent in the diseased groups, such as *Lachnoanerobaculum*, *Atopobium*, *Oribacterium, Peptostreptococcus*, *Rothia*, *Stomatobaculum*, *Klebsiella*, and *Prevotella*, showed much higher abundance in the latter groups, which might be highly correlated to the disease. Noticeably, *Serratia* was presented in 54.84% of CBDS samples, while absent in the HCs samples. Moreover, *Veillonella* showed a much higher occurrence frequency and average abundance in patients with bile duct diseases (i.e., CBDS and SCBD) (Table 2).

**Table 2:** Occurrence frequency and average abundance of each group's featured genera

Taxonomy	HCs Fre (Abun)^a^	GBS Fre (Abun)	CBDS Fre (Abun)	SCBD Fre (Abun)	LDA featured
*Atopobium*	25 (0.03)	73.33 (0.35)	80.65 (0.54)	87.50 (0.55)	SCBD
*BIyi10*	25 (0.002)	0 (0)	3.23 (0.0003)	0 (0)	HCs
*Bordetella*	100 (0.45)	46.67 (0.03)	35.48 (0.02)	62.50 (0.14)	HCs
*Brevundimonas*	100 (1.06)	66.67 (0.13)	64.52 (0.05)	100 (0.18)	HCs
*Castellaniella*	100 (0.93)	66.67 (0.45)	38.71 (0.05)	50 (0.62)	HCs
*Citrobacter*	100 (1.21)	66.67 (0.14)	61.29 (0.69)	87.50 (6.88)	SCBD
*Comamonas*	100 (1.30)	80 (0.72)	51.61 (0.51)	87.50 (0.67)	HCs
*Diaphorobacter*	100 (2.30)	66.67 (0.91)	54.84 (0.28)	62.50 (0.27)	HCs
*Lachnoanaerobaculum*	12.5 (0.006)	66.67 (0.12)	80.65 (0.30)	75 (0.37)	SCBD
*Ochrobactrum*	75 (0.84)	26.67 (0.005)	35.48 (0.008)	50 (0.03)	HCs
*Oribacterium*	12.5 (0.005)	73.33 (0.18)	80.65 (0.22)	75 (0.43)	SCBD
*Paracoccus*	100 (1.29)	53.33 (0.29)	58.06 (0.34)	50 (0.02)	HCs
*Parapusillimonas*	100 (2.68)	60 (0.07)	41.94 (0.07)	62.50 (0.42)	HCs
*Peptostreptococcus*	12.5 (0.005)	73.33 (1.63)	83.87 (0.25)	62.50 (0.14)	GBS
*Providencia*	87.5 (0.58)	53.33 (0.01)	29.03 (0.01)	62.50 (0.05)	HCs
*Pseudoalteromonas*	100 (2.02)	66.67 (0.21)	38.71 (0.09)	62.50 (0.34)	HCs
*Ralstonia*	100 (40.33)	66.67 (24.73)	51.61 (10.15)	75 (25.90)	HCs
*Rothia*	12.5 (0.02)	80 (1.75)	80.65 (0.18)	100 (0.21)	GBS
*Serratia*	0 (0)	20 (0.29)	54.84 (0.27)	25 (0.05)	GBS
*Stomatobaculum*	12.5 (0.004)	60 (0.06)	77.42 (0.27)	37.50 (1.79)	SCBD
*Subgroup_22*	87.5 (0.23)	40 (0.07)	25.81 (0.04)	37.50 (0.05)	HCs
*Vibrio*	100 (5.24)	66.67 (0.83)	58.06 (0.45)	62.50 (1.17)	HCs
*Klebsiella*	62.5 (0.07)	73.33 (1.51)	87.10 (6.66)	75 (0.67)	CBDS
*Prevotella*	75 (0.07)	80 (3.64)	87.10 (3.68)	87.50 (2.36)	CBDS
*Pseudomonas*	100 (11.16)	80 (4.65)	74.19 (5.23)	87.50 (12.01)	SCBD
*Veillonella*	87.5 (0.09)	73.33 (0.83)	90.32 (1.56)	87.50 (6.14)	SCBD
*Flavitalea*	25 (0.001)	0 (0)	0 (0)	0 (0)	HCs
*Gitt_GS_136*	25 (0.001)	0 (0)	0 (0)	0 (0)	HCs
*Humibacter*	0 (0)	0 (0)	0 (0)	25 (0.002)	SCBD
*Tistlia*	25 (0.0008)	0 (0)	3.23 (0.0003)	0 (0)	HCs

^a^Fre and Abun represent occurrence frequency and abundance, respectively. HCs, GBS, CBDS and SCBD represent Healthy Controls (liver donors without hepatobiliary disease), patients with Gall-Bladder Stones, patients with Common Bile Duct Stones, and patients with Stricture in the Common Bile Duct, respectively

### Correlations between co-occurrence taxa modules and clinical indexes

**Fig. 4:**
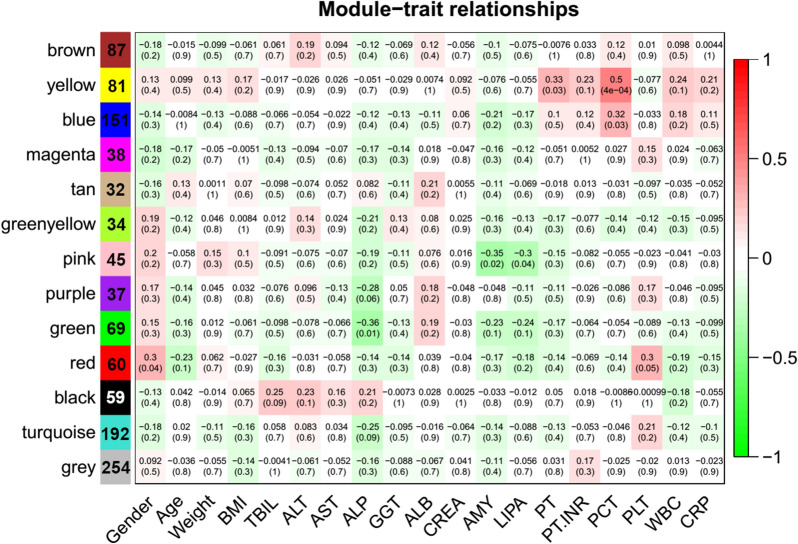
Correlations between the co-occurrence biliary taxa modules and clinical traits. The right color bar represents the module-trait correlation coefficient ranging from -1 to 1. The number in each cell of the left column represents the taxa counts those clustered in the corresponding module. The colors in the cells of the middle columns represent the correlation marked by the right color bar, and the numbers in the brackets are the *p*-values; the numbers outside the brackets are the correlation coefficients. Significant correlations were defined as *p*-values ≤ 0.05 and correlation coefficients > 0.3 or < -0.3. BMI, TBIL, ALT, AST, ALP, GGT, ALB, CREA, AMY, LIPA, PT, PT.INR, PCT, PLT, WBC and CRP represent body mass index, total bilirubin, alanine transaminase, aspartate transaminase, alkaline phosphatase, γ-glutamyl transpeptidase, albumin, creatinine, amylase, lipase A, prothrombin time, (prothrombin time)/ (international normalization ratio), procalcitonin, platelet, white blood cell and C-reactive protein, respectively

To decipher the correlations between the biliary bacteria and biliary diseases, Spearman’s correlation analysis was conducted between the alpha diversity and clinical indexes. The results showed that only five clinical indexes—namely age, AMY, LIPA, CRP, and WBC—significantly correlated with the alpha diversity, indicated by the Shannon–Weiner indexes (Additional file 2: Fig. S2). These results indicate that participants with acute pancreatitis after endoscopic surgery harbor a less diverse biliary bacterial community (indicated by the negative correlation between the AMY/LIPA pair and alpha diversity). The negative correlations between the CRP/WBC pair and alpha diversity suggest that inflammation can reduce the biliary bacterial diversity (Additional file 2: Fig. S2). Moreover, the clinical liver function index (TBIL, ALT, AST, ALP, and γ-glutamyl transpeptidase (GGT)), infection index (e.g., PCT), and inflammation index (e.g., WBC and CRP) were positively correlated (Additional file 2: Fig. S2). To further clarify the correlations between specific taxa groups and the clinical indexes, WGCNA was conducted using the bacterial abundance matrix as input data. Hierarchical clustering identified samples GBS1, GBS4, GBS6, CBDS11, CBDS13, and SCBD2 as outliers, which were removed during further analysis (Cutheight = 0.55) (Additional file 2: Fig. S3). The results show that a total of 1, 138 genera and species in the bile bacterial community could finally be separated into 13 taxa modules, colored brown, yellow, blue, magenta, tan, green–yellow, pink, purple, green, red, black, turquoise, and gray (Additional file 2: Fig. S4), with the counts of taxa in each module being 87, 81, 151, 38, 32, 34, 45, 37, 69, 60, 59, 192, and 254, respectively (Fig. 4) (Please see Additional file 1: Table S2 for details). Among them, modules yellow and blue were positively correlated with PCT, module pink was negatively correlated with AMY and LIPA, and module green was negatively correlated with ALP (Fig. 4). These results suggest some taxa clusters are highly correlated to clinical indexes, such as PCT, AMY, and LIPA. Specifically, bacteria in module yellow, including *Insolitispirillum*, *Haloferula*, *Balneola*, *Fermentibacteraceae*, *Cellvibrionaceae*, and *Plesiomonas*, displayed high correlations (> 0.8) with the microbial infection index PCT (Additional file 1: Table S2). Collectively, the variations of some specific bacteria may result in biliary system infections or inflammation, thus leading to poor outcomes after endoscopic surgery.

### Specific biliary bacteria correlated with poor outcomes after endoscopic surgery

**Fig. 5:**
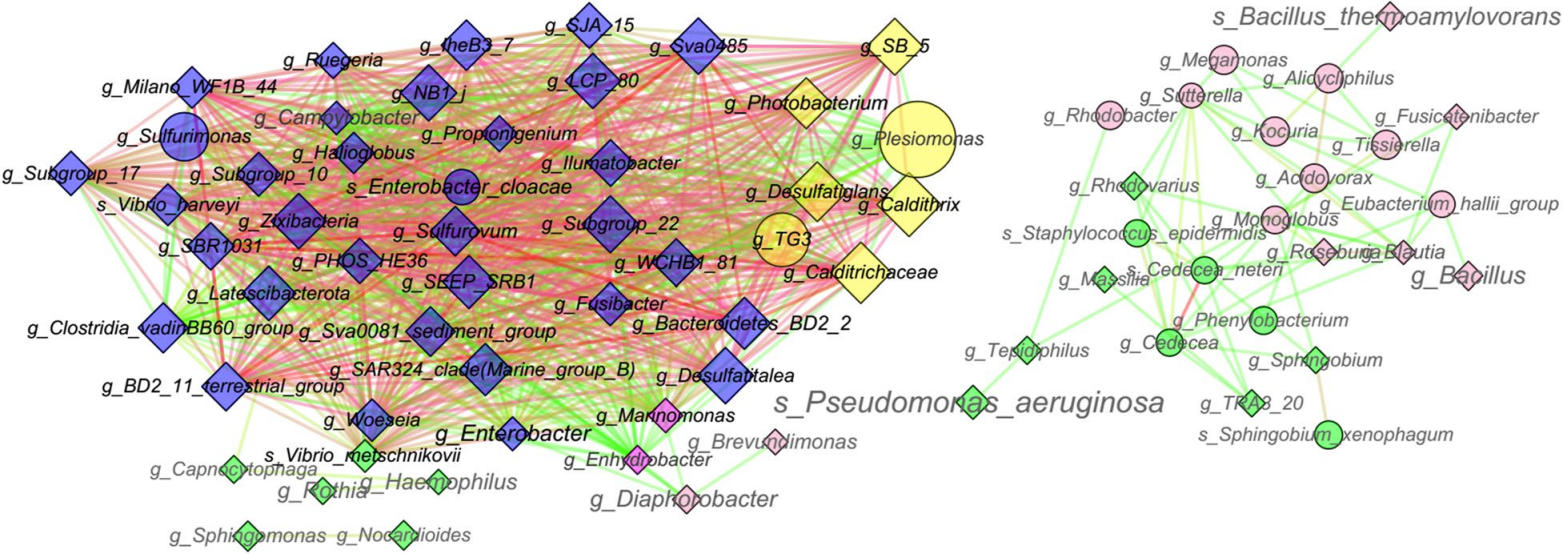
Networks visualization of the clinically relevant taxa modules. Only taxa with a correlation coefficient higher than 0.5 or lower than − 0.5 are displayed (*p*-value ≤ 0.05). The different node colors (i.e., yellow, blue, green, magenta, and pink) in the networks represent different genus modules. The node label size in the network represents the average abundance of the taxa; the bigger the node label size, the more abundant the taxa. The node label color represents the network connectivity of the taxa; the darker the color, the higher connectivity it represents. The node size represents the correlations between the taxa and PCT; a bigger node size indicates higher correlations. The color of the network edge represents the correlations between the taxa; the green color represents low correlations while the red color represents high correlations. The node shape represents different ecological categories of the taxa, from which diamond represents intermittent taxa and eclipse represents transient taxa

The co-occurrence networks of clinical correlated taxa modules are displayed in Fig. 4. Seven genera in module yellow, namely *SB_5*, *Photobacterium*, *Plesiomonas*, *Desulfatiglans*, *Caldithrix*, *TG3*, and *Calditrichaceae* were highly positive-correlated with PCT, indicating potential microbial infections (Fig. 5). Among them, *Plesiomonas* represented the most possible pathogen because it showed the highest correlation with PCT (Additional file 1: Table S2), as well as high connections in the co-occurrence network (Fig. 5). Moreover, the taxa in module blue are highly correlated with the taxa in module yellow, and showed medium correlation with PCT. Noticeably, some taxa in modules green and pink are negatively connected with the taxa in module yellow in the networks, such as *Vibrio_metschnikovii*, *Enhydrobacter*, *Brevundimonas*, and *Diaphorobacter*, and have been identified as biomarkers in HCs using LEfSe analysis (Fig. 3). This indicates that the reduction of these taxa may be potential diagnostic biomarkers between the HCs and biliary diseased patients (e.g., CBDS, SCBD, and GBS). Other taxa identified as biomarkers in the different groups using LEfSe have also been clustered into modules pink and green, which negatively correlated with ALP or AMY/LIPA, indexing biliary system inflammation and acute pancreatitis. For example, *Bacillus* and *Pseudomonas* were identified as biomarkers in SCBD, which clustered into module pink and module green, respectively, and were negatively correlated with AMY/LIPA and ALP (Fig. 4). Furthermore, all taxa in the networks are intermittent or transient, implying that the frequent presence or absence of specific bacteria occurred in the diseased gallbladder and poor outcomes after endoscopic surgery may result from alien bacterial contamination (e.g., *Photobacterium* and *Plesiomonas*) or local bile bacteria loss (e.g., *Brevundimonas* and *Diaphorobacter*) during the surgery.

## Discussion

Gallstone disease and its endoscopic treatment may result in several complications that severely lower the quality of life of the patient; the progression of the disease can even contribute to biliary cancers [32]. Bacterial infections are common complication causatives during the disease or endoscopic treatment process. In this study, we compared bile bacterial communities between the HCs and three groups of gallstone patients at different pathological states (i.e., GBS, CBDS, and SCBD). The results show that the bile harbors a relatively stable bacterial community as no significant differences were observed in the alpha diversity or beta diversity between each group from a global view. Of note, high heterogeneity was observed in the CBDS group and specific taxonomic variations existed between each group. The biliary taxa abundance and occurrence frequency were found to fit exponential correlations, and the majority of the taxa were intermittent or transient and low in abundance. The featured taxa identified using LDA in each group were mainly intermittent or transient taxa, among which *Lachnoanerobaculum*, *Atopobium*, *Oribacterium, Peptostreptococcus*, *Rothia*, *Stomatobaculum*, *Klebsiella*, and *Prevotella* showed much higher presence in the diseased groups. Moreover, patients with acute pancreatitis after endoscopic surgery displayed lower alpha diversity. Although some taxa, such as *Photobacterium*, *Plesiomonas*, *Desulfatiglans* and *Caldithri*, were not featured in any group using LDA, they were co-clustered and highly positive-correlated with microbial infection (indicated by PCT) after endoscopic surgery. However, some LDA-featured taxa in HCs, such as *Vibrio metschnikovii*, *Enhydrobacter*, *Brevundimonas*, and *Diaphorobacter*, were co-clustered and negatively correlated with biliary system inflammation and acute pancreatitis.

Studies in recent years have confirmed that the biliary system harbors a complex microbiota; among them, Proteobacteria, Firmicutes, Bacteroidetes, Fusobacteria, and Actinobacteria are the most predominant phyla described in both the healthy and diseased biliary bacterial communities by many studies [23, 33, 34]. Similar to earlier reports, these phyla were most abundant in bile in this study, especially for Proteobacteria that had 60% abundance, which showed identical abundance to that described in the small intestine [35]_._ For the first time, Molinero et al. [23] compared the bacterial communities between gallstone/cholelithiasis patients and individuals without any hepatobiliary pathology (i.e., liver donors), and observed that Proteobacteria were more abundant in the control group while Bacteroidota were more abundant in the cholelithiasis group at the phylum level; and a significantly higher abundance of *Escherichia-Shigella* was observed in the cholelithiasis group at the genus level. Similar bacterial variations between healthy controls and disease groups have also been observed in this study. For example, Proteobacteria was found to be the most abundant taxon in the control group, while Firmicutes and Bacteroidota were the most abundant in the CBDS group (Fig. 1e). At the genus level, *Ralstonia* was found to be the most abundant genus in the control group and its abundance decreased over 14% in the diseased groups (Fig. 1f). Previous studies have demonstrated that certain bacteria can work cooperatively by encoding specific enzymes or macromolecules, thereby facilitating gallstone formation through the synthesis of essential components [8]. For example, *Clostridium perfringens* and *E. coli* have been found to produce beta-glucuronidase, which releases free bilirubin from the bilirubin diglucuronide, the free bilirubin then precipitates with free ionized calcium ion to form calcium bilirubinate, a major component of pigment gallstones; the lipopolysaccharides produced from Gram-negative bacteria, such as *Escherichia* sp., *Klebsiella* sp., and *Pseudomonas* sp., can stimulate mucin secretion from dog gallbladder epithelium, which functions as an integrating factor to coagulate amorphous material into gallstones [8, 36]. We observed in this study that *Escherichia-Shigella* and *Klebsiella* were far more abundant while *Ralstonia*, *Lactobacillus*, and *Neisseria* were far less abundant in the CBDS patients than those in the GBS patients, indicating that with the gallstones migrating from the gallbladder to the common bile duct, the abundance of specific taxa greatly changed. Interestingly, with the disease progression, some taxa related to gallstone formation, such as *Escherichia-Shigella* and *Klebsiella*, also got increased. To the best of our knowledge, this is the first study to explore the bacterial composition differences between GBS and CBDS. In several studies, alpha diversity of the bile bacteria decreased significantly in the diseased group, and a divergent bacterial community was observed between the groups. For example, Molinero et al. reported a decreased alpha diversity in the cholelithiasis group and divergent bacterial communities between the healthy and cholelithiasis groups (indicated by beta diversity) [23]. Dangtakot et al. [14] found a decreased bacterial richness and increased diversity between CBDS and cholangiocarcinoma (CCA) patients. However, paradoxical results exist as no significant differences were observed in the alpha diversity or beta diversity between each group in this study and some other studies. For example, Kim et al. [33] recently reported that no significant differences existed between CBDS, benign biliary stricture, gallbladder cancer, pancreatic cancer, and distal cholangiocarcinoma (dCCA) in the bile microbiota. Chen et al. [34] observed similar bile bacterial richness between patients with dCCA and the new onset CBDS. Pereira et al. [21] revealed no significant differences in bacterial communities between early-stage primary sclerosing cholangitis (PSC) patients and non-PSC patients_._ In comparison to the extensive studies, this paradox reminds us that more efforts are needed to decipher the bile microbiota, particularly those taking healthy bile samples (e.g., liver donors) as controls. In contrast, high heterogeneity was observed in CBDS and SCBD when compared with GBS or HCs, which was also observed in a previous study on patients with common bile duct stones (bile was collected via ERCP) [34], suggesting that strict standards should be kept in the ERCP surgery to reduce bacterial contamination from the intestine. Furthermore, clinical indexes relevant to the biliary pathological state, especially for microbial infection and acute pancreatitis, should be highly important and taken into consideration to obtain more creditable results concerning the involvement of the microbiota in these diseases.

Current studies investigating bile bacteria alterations between different pathological states are devoted to describing taxa abundance or diversities, which may underestimate the importance of the occurrence frequency of specific taxa between different groups. Previous studies have reported that the majority of taxa are low in abundance while the minority are high in abundance in a specific ecosystem. Zhang et al. [37] reported that 25.5% of the top abundant genera accounted for 89.1% of the cumulative abundances in the microbial communities of activated sludge from 14 wastewater treatment plants across Asia and North America. This microbiota assemblage pattern has also been observed in other studies [38]. In this study, the top 9.52% and 18.63% abundant taxa showed 89.41% and 84.71% accumulative abundances at the phylum and genus levels, respectively, indicating that the bile microbiota assembly pattern was similar to other ecosystems. Moreover, the best model of taxa abundance and occurrence frequency in the bile was calculated to be fitting for the exponential correlations, which have been found in other communities [29, 37]. The LEfSe analysis indicates that the most featured taxa between the different pathological groups were intermittent or transient. The only highly represented taxa in CBDS were two persistent taxa (i.e., *Klebsiella* and *Prevotella*). Furthermore, two persistent genera (i.e., *Pseudomonas* and *Veillonella*) are highly represented in SCBD. In previous studies, *Klebsiella* increased significantly in CBDS when compared with the healthy group and was associated with severe cholangitis [22, 39]. *Prevotella* was previously identified as the dominant biliary genera in gallstone patients and aligned with salivary microbiota [34, 40], which was also a pathogen causing chronic endodontic infection [41]. In another study, *Pseudomonas* was more abundant in CCA than that in gallstone patients [14]. Although these four taxa were frequently enriched in the diseased biliary bacterial community, in this study, they were persistent between the healthy and diseased groups (Table 2), which makes it more possible for them to be the results of the biliary diseases but not the drivers.

Alongside gallstone disease progression, some persistent and harmless taxa in the bile may become pathogenic. For example, *Veillonella* is an opportunistically pathogenic commensal commonly found in the oral, genitourinary, respiratory, and intestinal tract of humans, while it may cause various rare infections in immunocompromised host [42]. Moreover, *Veillonella* was found increased in PSC patients when compared with that in HCs [43]. Our study identified *Veillonella* as a persistent bacterium in the biliary bacterial community, and its abundance increased sequentially alongside HCs→GBS→CBDS→SCBD (Table 2). While it remains speculative to propose that *Veillonella* becomes pathogenic during disease progression, further investigation is warranted to explore this possibility. In contrast, six LDA featured genera that were transient or intermittent in HCs but persistent in the diseased groups have also been reported in previous studies. Interestingly, five of them were abundant and frequently isolated in the oral cavity. For example, *Lachnoanerobaculum* has been isolated from human saliva and found to induce bacteremia in a patient with leukemia [44]. *Atopobium* increased in patients with pancreatic cancer [45], and was frequently isolated from the oral cavity [46]. *Rothia*, aligned more with the salivary microbiota of patients, increased in patients with pancreatic cancer, and was dominant in patients with gallstone diseases [46, 47]. *Oribacterium* in the oral cavity has been suggested as a biomarker for pediatric-onset PSC diagnosis or pancreatic head cancer [48]. *Stomatobaculum* in the saliva was correlated with gastroesophageal reflux disease [49]. In addition, *Peptostreptococcus*, which resides in the intestine, participated in the oxidation and epimerization of bile acids and led to the formation of gallstones, and may be potentially involved in gallbladder cancer progression [32]. Noticeably, the abundances of four out of the five oral cavity-aligned taxa (i.e., *Lachnoanerobaculum*, *Atopobium*, *Oribacterium*, and *Stomatobaculum*) increased sequentially during disease progression (i.e., HCs→GBS→CBDS→SCBD) (Table 2), and belonged to the phyla Firmicutes and Actinobacteriota (Additional file 1: Table S3). Collectively, bacteria translocation from the oral cavity may play a vital role in gallstone formation and progression, which could be developed as effective diagnostic biomarkers for biliary disease or indicators for disease progression (Additional file 2: Fig. S5). The overgrowth or undergrowth of persistent bacteria within the biliary system could also contribute, but they are less likely to be the drivers. In the future, strain-level taxonomic assignment analysis should be conducted to further confirm the underlying pathogenic mechanisms of these featured taxa.

Under clinical conditions, poor outcomes of endoscopic surgery, such as pancreatitis and microbial infections frequently occur, although antibiotics are administered before the procedure. To our knowledge, the variations in the bile bacterial community between good outcomes and poor outcomes have not been explored. Using Spearman’s correlation analysis, acute pancreatitis after endoscopic surgery was found to correlate with lower bacterial diversity (Additional file 2: Fig. S2). This result reminds us that when comparing bile bacterial community variations between different diseased groups, the clinical indexes should be taken as parameters for grouping. We further found that the specific taxa that were mostly correlated with poor outcomes were not featured by LDA score between the disease groups (Fig. 3 and Fig. 5). This result implies that although some taxa were increased or decreased significantly in the diseased groups, they are not the cause of poor outcomes after endoscopic surgery. Of note, among the module yellow taxa, which are mostly correlated with poor outcomes, two (*Photobacterium* and *Plesiomonas*), have been identified as human pathogens causing wound tissue and intestinal infections [50–52], while there is no evidence of the pathogeny of the other five; however, they should be critically considered when acute pancreatitis or microbial infections are observed after endoscopic surgery. For the taxa presenting 100% in HCs (*Pseudomonas*, *Brevundimonas*, and *Diaphorobacter*) and featured in HCs by LDA score, their loss in the diseased biliary system may result in a higher probability of developing acute pancreatitis after endoscopic surgery, as they are negatively correlated with ALP or AMY/LIPA. As indicated in Fig. 5, the majority of the taxa that positively correlated with infections are intermittent taxa, which may be contaminations from the upper digestive tract or small intestine during the endoscopic surgery process.

In conclusion, we compared the bile bacterial community variations between different pathological states in gallstone disease progression and revealed that the bile harbors a relatively stable bacterial community during disease progression. However, specific taxa variations were observed between the HCs, benign gallstone disease patients, and malignant gallstone disease patients. A few taxa consist of the core taxa in the bile, accounting for most of the abundance. Moreover, exponential correlations exist between bacterial abundance and occurrence frequency. Notably, abundance alteration of the persistent taxa may not be the cause of gallstone formation and progression, while bacterial translocation and colonization from the oral cavity plays a vital role. Furthermore, the bacterial group that causes infection or inflammation after endoscopic surgery is not the same as that correlated with disease development or progression and may result in a reduction in biliary bacterial diversity. This study extends our knowledge of potential bacterial contributions to gallstone disease progression and outcomes after endoscopic surgical treatment. However, limitations exist because of the low sampling volume and non-strain level taxa assignment, and some of the generated results were speculative, which warrants further studies.

### Supplementary Information

**Additional file 1: Table S1.** Quality data of the 16S rRNA amplicon sequencing. **Table S2.** Bacterial taxa modules and their correlations to clinical indexes. **Table S3.** Taxonomic characterization of the taxa that sequentially increased in the bile of HCs, GBS, CBDS and SCBD.

**Additional file 2: Figure S1.** Rarefaction curve displayed using Shannon diversity (**a**) and accumulated ASVs (**b**). **Figure S2.** Correlations between the clinical traits and alpha diversity. Correlations significant at different levels were marked as follows: *p-value ≤ 0.05, **p-value ≤ 0.01, ***p-value ≤ 0.001. **Figure S3.** Hierarchical clustering to determine outlier samples. Samples GBS1, GBS4, GBS6, CBDS11, CBDS13 and SCBD2 were identified as outliers. Red line on the tree indicates the cut-height to divide clusters (Cutheight=0.55). **Figure S4.** Co-exist taxa module identified using dynamic tree cut method implanted in the WGCNA package. Each color represents one taxa module. **Figure S5.** Scheme displaying oral cavity bacteria translocation with gallstone disease progression.

## Data Availability

The raw reads datasets generated for this study can be found in the Short Read Archive (SRA) database: https://www.ncbi.nlm.nih.gov/sra/PRJNA856088.
