# Supplementary material for: Variations of bile bacterial community alongside gallstone disease progression and key taxa involved in poor outcomes after endoscopic surgery
Source: Eur J Med Res. 2023 Sep 2;28:313. doi: 10.1186/s40001-023-01308-y (PMC10474685; doi:10.1186/s40001-023-01308-y)
Supplement: Supplementary file 2 — Additional file 2: Figure S1. Rarefaction curve displayed using Shannon diversity (a) and accumulated ASVs (b). Figure S2. Correlations between the clinical traits and alpha diversity. Correlations significant at different levels were marked as follows: *p-value ≤ 0.05, **p-value ≤ 0.01, ***p-value ≤ 0.001. Figure S3. Hierarchical clustering to determine outlier samples. Samples GBS1, GBS4, GBS6, CBDS11, CBDS13 and SCBD2 were identified as outliers. Red line on the tree indicates the cut-height to divide clusters (Cutheight=0.55). Figure S4. Co-exist taxa module identified using dynamic tree cut method implanted in the WGCNA package. Each color represents one taxa module. Figure S5. Scheme displaying oral cavity bacteria translocation with gallstone disease progression. [file 40001_2023_1308_MOESM2_ESM.docx]

**Variations of Bile Bacterial Community Alongside Gallstone Disease Progression and Key Taxa Involved in Poor Outcomes After Endoscopic Surgery**

Xunchao Cai^1, †^, Yao Peng^1, †^, Yajie Gong^1^, Xiuting Huang^1^, Lu Liu^1^, Yifan Chen^1^, Jingfeng Du^1^, Zhongming Dai^1^, Yun Qian^1^, Long Xu^1, 2*^.

^1^ Department of Gastroenterology and Hepatology, Shenzhen University General Hospital, 518055 Shenzhen, China

^2^ Marshall Laboratory of Biomedical Engineering, Shenzhen University, 518055 Shenzhen, China

^†^ These authors contributed equally to this study and share first authorship.

**^*^Correspondence to:**

Long Xu, Department of Gastroenterology and hepatology, Shenzhen University General Hospital, 518055 Shenzhen, China. Email: longxu1012@szu.edu.cn.


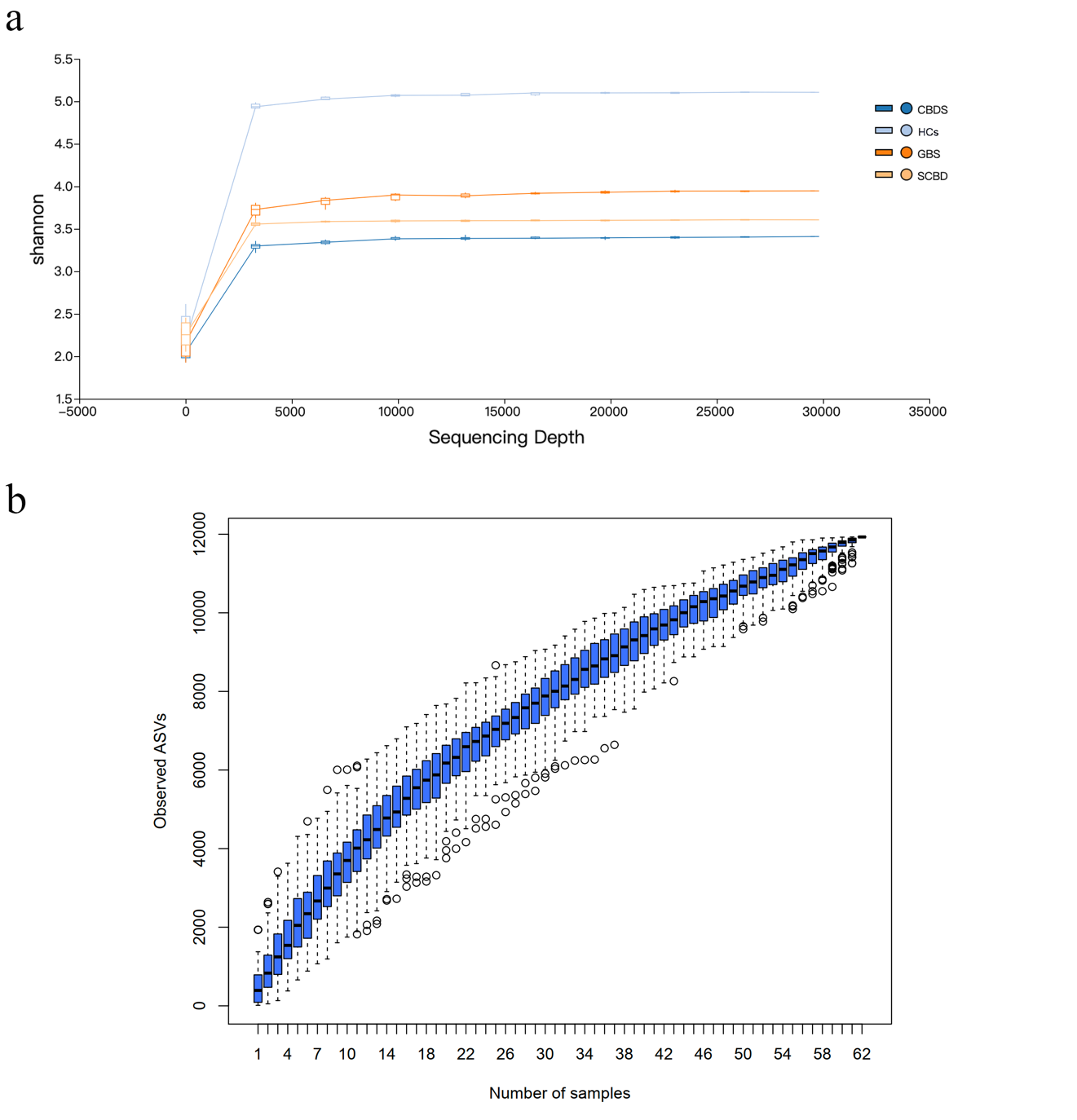


Figure S1 Rarefaction curve displayed using Shannon diversity (a) and accumulated ASVs (b).


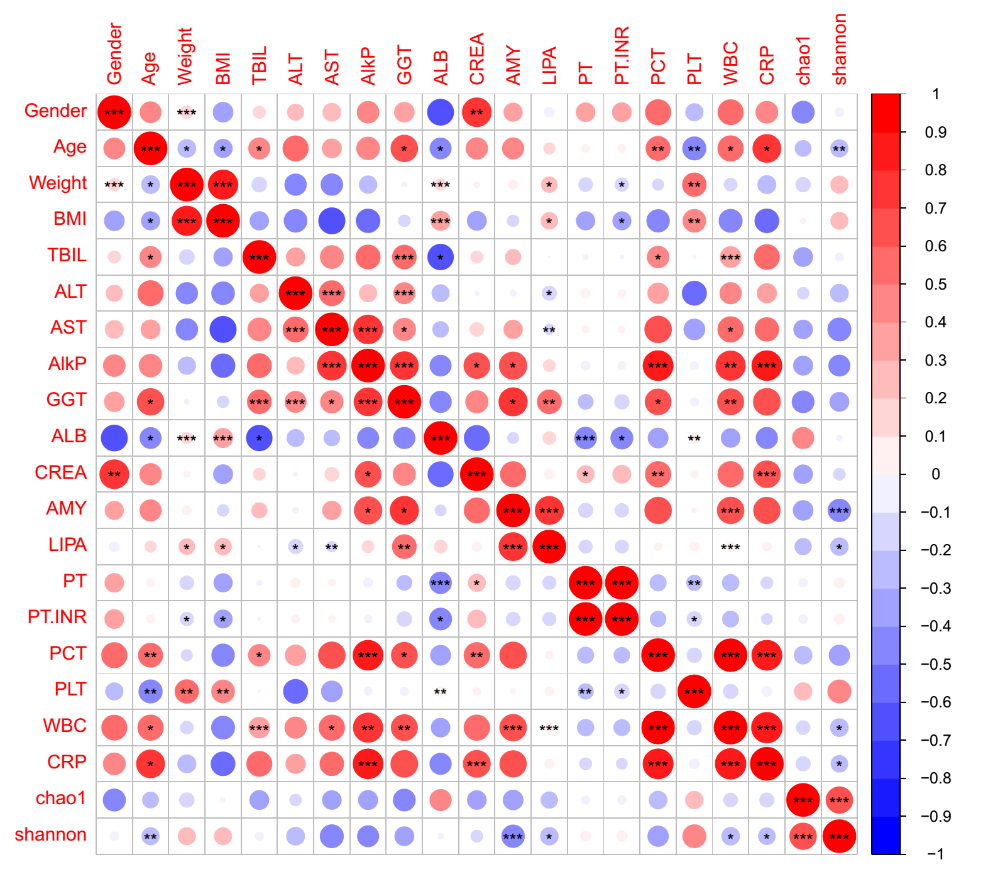


Figure S2 Correlations between the clinical traits and alpha diversity. Correlations significant at different levels were marked as follows: **p*-value ≤ 0.05, ***p*-value ≤ 0.01, ****p*-value ≤ 0.001.


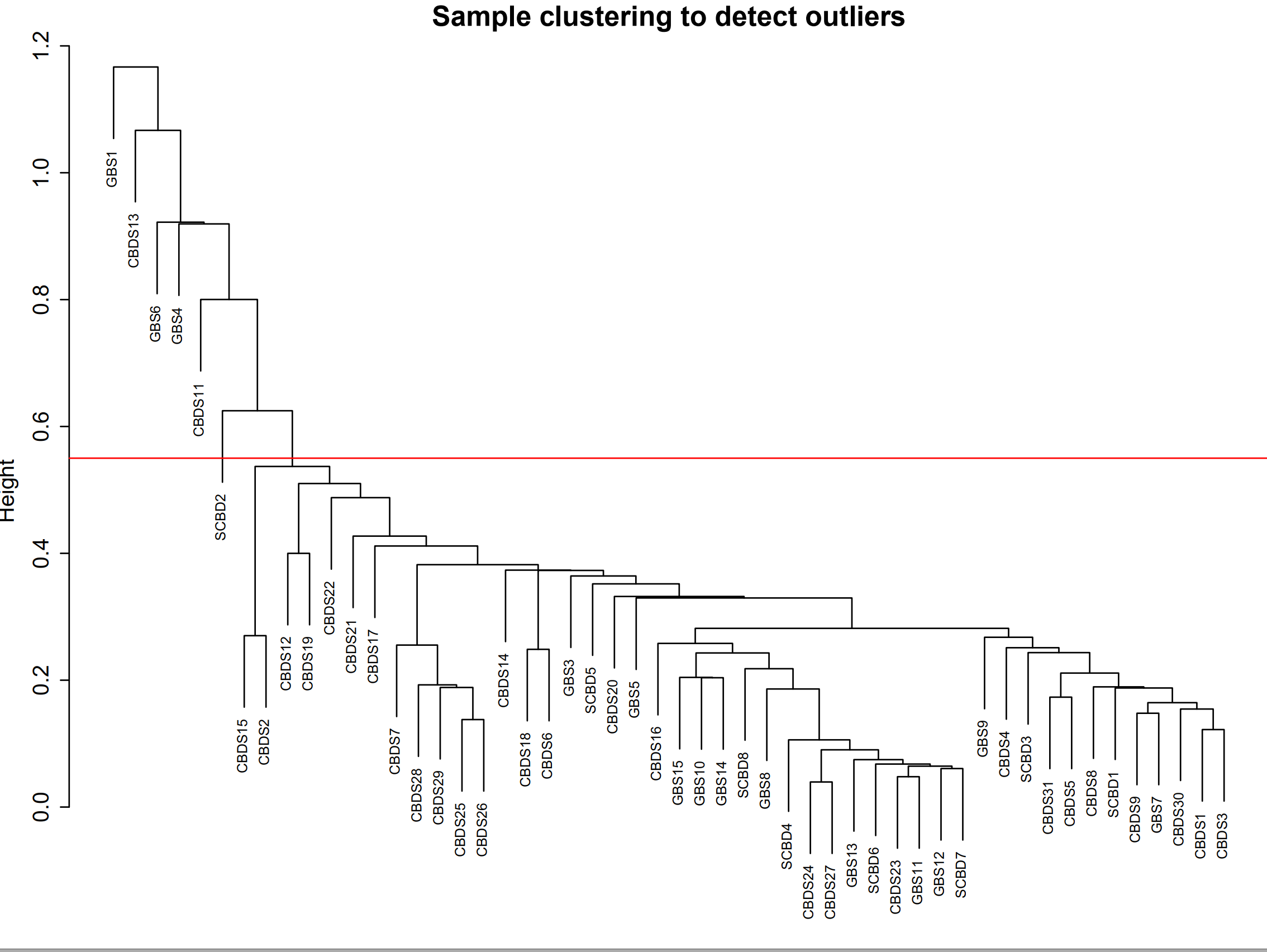


Figure S3 Hierarchical clustering to determine outlier samples. Samples GBS1, GBS4, GBS6, CBDS11, CBDS13 and SCBD2 were identified as outliers. Red line on the tree indicates the cut-height to divide clusters (Cutheight=0.55).


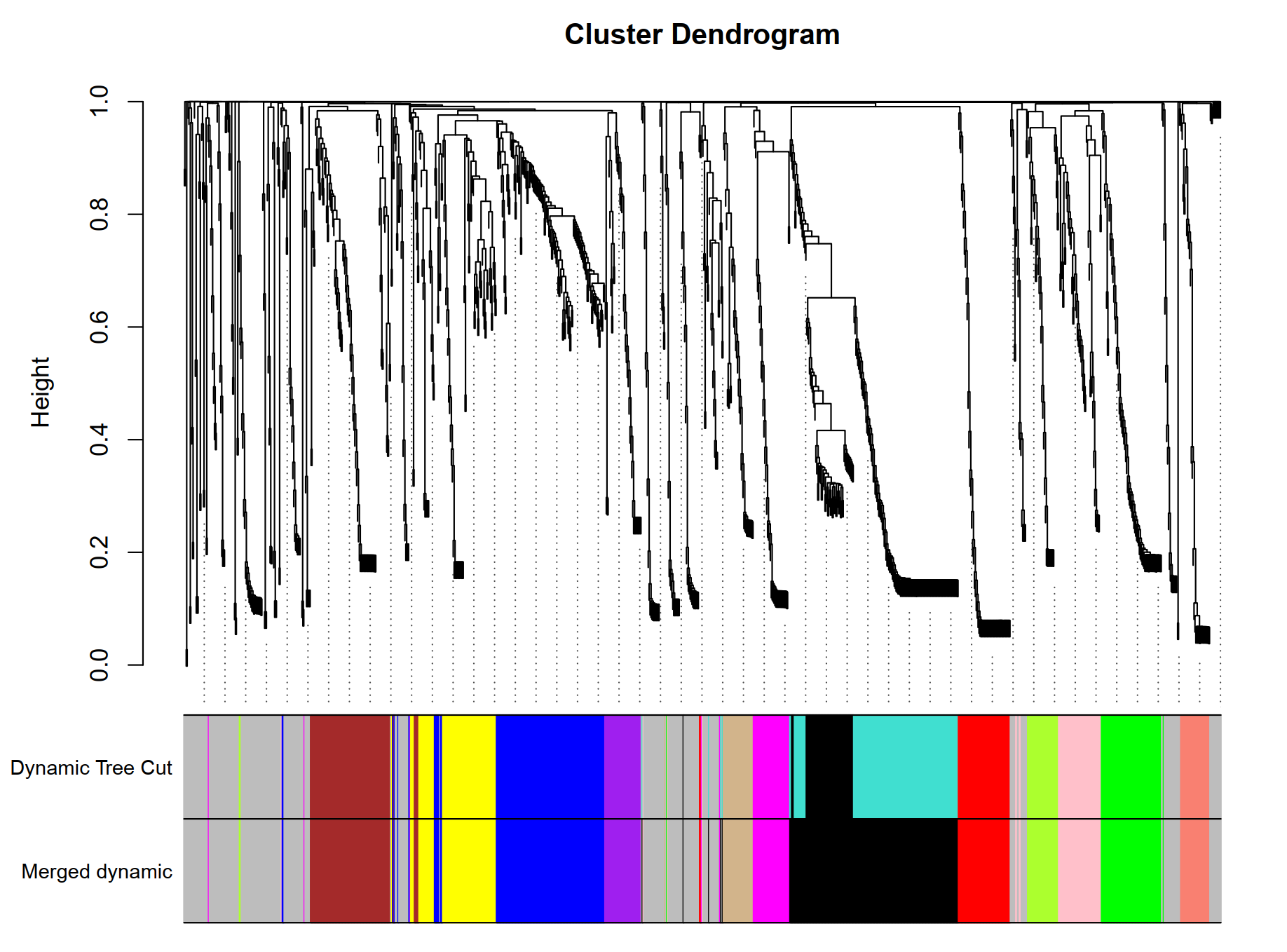


Figure S4 Co-exist taxa module identified using dynamic tree cut method implanted in the WGCNA package. Each color represents one taxa module.


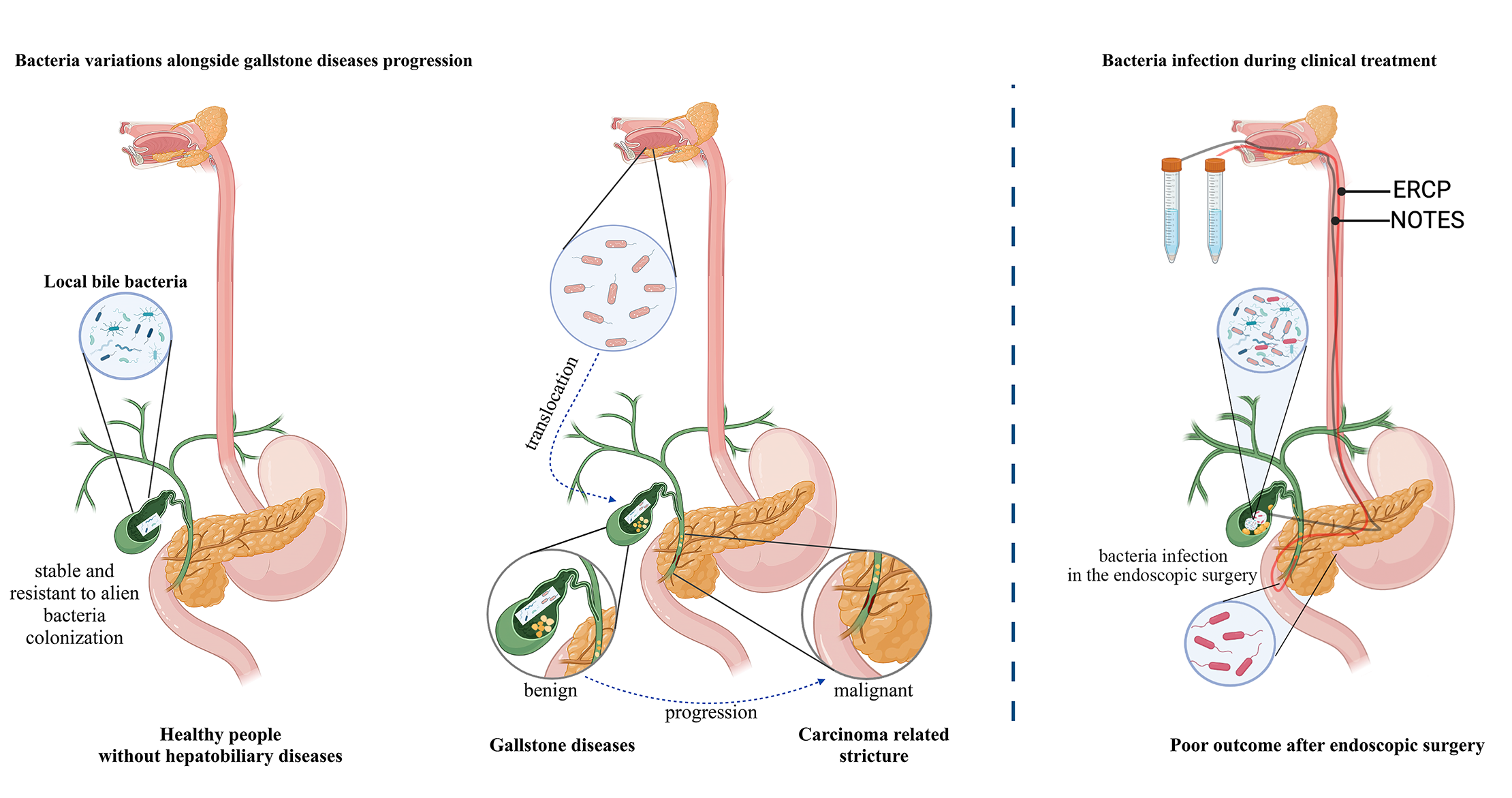


Figure S5 Scheme displaying oral cavity bacteria translocation with gallstone disease progression.
